# L-Carnitine reduces the negative effects of formalin on sperm parameters, chromatin condensation and apoptosis in mice: An experimental study

**DOI:** 10.18502/ijrm.v13i10.7768

**Published:** 2020-10-13

**Authors:** Daniyal Ezati, Reyhane Vardiyan, Ali Reza Talebi, Morteza Anvari, Majid Pourentezari

**Affiliations:** ^1^Department of Biology and Anatomy, Shahid Sadoughi University of Medical Sciences, Yazd, Iran.; ^2^Research and Clinical Center for Infertility, Yazd Reproductive Sciences Institute, Shahid Sadoughi University of Medical Sciences, Yazd, Iran.

**Keywords:** Formalin, L-carnitine, Mice, Sperm chromatin, Apoptosis.

## Abstract

**Background:**

Formalin is commonly applied as an antiseptic and tissue fixative. It has reactive molecules that lead to its cytotoxic effects. According to recent studies, formalin causes a change in the testicular and sperm structure and L-carnitine (LC) acts as an antioxidant to counteract its effects.

**Objective:**

This study aimed to investigate the protective effects of LC on the parameters, chromatin condensation and apoptosis of mice sperm exposed to formalin.

**Materials and Methods:**

In this experimental study, 24 balb/c mice (25-40 gr,10-12 wk) were divided into three groups (n = 8/each): group I without any injections or gavage; group II, received 10 mg/ kg formalin intraperitoneally (I.P); and group III was exposed to formalin and LC, where a dose of 10 mg/kg formalin was injected I.P daily and LC the dose of 100 mg/kg was kept in a solvent solution. After 31 days, the sperm examination was performed as follows: to evaluate chromatin and DNA quality of the sperm, we applied aniline blue (AB), toluidine blue (TB), chromomycin A3 (CMA3), and terminal transferase-mediated deoxy uridine triphosphate biotin end labeling (TUNEL) tests.

**Results:**

Sperm parameters such as count, motility, morphology, and viability displayed a significant decrease in the formalin group. While the data exhibited a considerable augment in sperm parameters in the formalin + LC than the formalin and control groups (p < 0.001), significant differences were detected between groups with respect to TB staining, TUNEL test, CMA3 test and AB staining in the formalin and formalin + LC groups.

**Conclusion:**

LC can reduce the negative effects of formalin on sperm parameters, chromatin stability, and percentage of apoptosis in an animal model.

## 1. Introduction

While infertility is generally supposed to be a female problem, male-factor infertility is obvious in 50% of infertile pairs, with 30-40% of these cases being due to sperm abnormalities (1). Several factors are related with male infertility, containing an injury in the genital organ hurt, varicocele, seminal fluid infection, reproductive duct obstruction, endocrine and metabolic disease, and environmental factors such as alcohol intake and cigarette smoking (2). One of the experiment has revealed evidence of the damaging effects of environmental pollutants on male fertility (3). Formaldehyde (FA, CH2O) is a combustible, uncolored, and bitter chemical factor that is applied extremely in manufactories, hospitals, technological laboratories, and households (3, 4). It is known as indispensable chemical mixtures in the worldwide economic system. Extensive utilization of FA has augmented its environmental or vocational exposure (5). The generative system is adversely affected by FA, so that it declines sperm parameters like count, viability, motility, and morphology of sperm (6, 7). Facing with FA leads to testicular disturbance by reducing antioxidant enzymes performance and promoting lipid peroxidation, consequently stimulating the oxidative stress in testes (8, 9). FA also harms male germ cells and elevates transcription of Hsp70 and Bax gene in testis (10, 11). Increasing the DNA-protein crosslinks is the key pathway of FA toxicity that results in the activation of p53 and pro-apoptotic gene expression (12, 13). Regarding the results of abundant research, the use of antioxidants is able to inhibit the cellular damage caused by oxidation as well as improve of the sperm quality and genital operation (14, 15). Therefore, research into drugs or materials to reduce the side effects of formalin on the male reproductive system is necessary. L-Carnitine (LC) demonstrates a key role in the oxidation of long-chain fatty acid, and its active form, L-acetyl carnitine (ALC), supports mitochondria from metabolic toxins with its antioxidant factors. ALC also reinstates cell membranes and performs antiapoptotic actions (16). Moreover, LC absorbed by epididymal cells is freed into the epididymal lumen of the seminiferous epithelium. The condensation of LC in the epididymal lumen is nearly 2,000-fold greater than in the blood circulation, indicating that it plays a very important role in sperm parameters and metabolism (17).

Besides, LC also has some beneficial effects on spermatogenesis, sperm maturation, and sperm motility (18). It is a tiny ammonium compound involved in lipid metabolism in mammalian (19) It has also been revealed that LC and related compounds have antioxidant and anti-inflammatory impacts on several physiological situations (20). Meanwhile, it has been determined that LC can serve as a crucial antiapoptotic agent (21). Moreover, LC elevates the function of DNA-repairing enzyme as well as other associated repair pathways (22). The usage of LC and related compounds is a novel approach for improving fertility in humans. Recently, human and animal experiments have specified a potential role for using carnitine as an antioxidant and free radical sweeper that are able to raise seminal fluid quality. In addition, the pathway that carnitines regulate male fertility is not yet well-known and they may lead to several disorders such as nausea, vomiting, stomach upset, seizures, diarrhea, and heartburn (23, 24). Our previous experiment indicated that formalin has an adverse impact on sperm parameters and chromatin stability in mice.

This study was planned to assess the impacts of LC on the chromatin compaction and the percentage of apoptosis in male balb/c mice treated by formalin.

## 2. Materials and Methods

### Animals and care

In this experimental study, twenty four adult balb/c mice (25-40 gr, 10-12 wk) were divided into three groups (n = 8/each): The control group (I) did not receive any injections or gavage; the formalin group (II) received 10 mg/kg formalin (25) intraperitoneally (I.P.); and the third group (III) was exposed to formalin and LC, injected I.P. daily with a dose of 10 mg/kg formalin, and the dose of 100 mg/kg LC (26) was kept in a solvent solution. The mice were kept in isolated cages for 31 days (almost a spermatogenesis period) and transmitted into a controlled location with a temperature ranging 25 ± 3°C and mean average moisture of 50 ± 5%. They were fed “mice chow” and the water was available for them.

### Epididymal sperm provision

Mice in all groups were anesthetized via ketamine and xylazine (10 mg/kg and 12 mg/kg, respectively) and their separated cauda epididymis was transmitted in 1 ml of pre-warmed Ham's F10. Temperate rapturing was accomplished to swim-out spermatozoa into a culture medium and the containers were kept in the incubator (at 37°C, 5% CO${}_{2}$) for about 15 min (27).

### Sperm examination

Sperm parameters were appraised for about 200 spermatozoa per animal. The sperm count and motility were measured using Maklr chamber. Motility was represented as the percentage of progressive, non progressive, and immotile sperm. Sperm viability and morphology were assessed via Eosin and Diff Quick staining tests, respectively (28).

### Chromatin stability valuations

All the dyestuffs and reactants were bought from Sigma Aldrch Company (St Lous, MO, USA). The efficiency of dyestuffs was examined with and without acid denaturation of some sperm samples and they were accepted as positive and negative controls, respectively (28).

### Aniline blue (AB) staining

Aniline blue selectively stains lysine-rich histones and is able to show those sperm chromatin condensation anomalies that are related to residual histones. (29). A semen stain was outspread on the glass slides to dry out in the open air. In brief, all the smears (derived from washed semen samples) were fixed in 4% buffered glutaraldehyde for 35 min at 20-25°C. All smears were stained with 6% aqueous AB stain and blended with 5% acetic acid (pH = 3.4) for 8 min. The three categories of head staining concentrations were specified: unstained or gray/white stained (standard spermatozoa or AB-) and complete sperm head stained dark blue (atypical spermatozoa, AB+).

### Toluidine blue (TB) staining

To perform this staining, after the smears were dried out in the open air, they were fixed in freshly made 95% ethanol-acetone (1:1) at 5°C for 35 min and then hydrolyzed in 0.2 NHCl at 5°C for 8 min. The slides were cleaned in three changes of distilled water for 3 min and eventually stained with 0.06% TB in 60% McIlvaine buffer (pH = 3.6) for 15 min at 20-25°C. The chromatin stability of spermatozoa was specified pursuant to metachromatic staining of sperm heads applied under a light microscopy at ×1000 eyepiece magnification (30, 31). Sperm heads with undamaged chromatin were light blue (TB${}^{-}$), those with mild abnormal chromatin were dark blue (TB+), and those with severely damaged chromatin and atypical packaging were deep violet and purple (TB+). About 200 sperm per slide were counted to compute the quantity of sperm with blue and violet head (32).

### Chromomycin A3 staining

Spermatozoa recovered from washed sample and semen smears were fixed in Carnoy's solution at 5°C for 15 min. Each slide was treated with 160 ml of CMA3 (0.30 mg/ml) in McIlvan buffer for 25 min. The slides were cleaned in buffer and mounted with buffered glycerol. Fluorescence was executed via microscope. The spermatozoa were assessed in each sample. Two kinds of staining template were determined: bright yellow-stained (atypical chromatin packaging) and yellowish green-stained (standard chromatin packaging) (29).

### Assessment of sperm apoptosis by TUNEL assay

The samples were fixed with methanol 100% (4 min) and incubated them in a blocking solution (3%H${}_{2}$O${}_{2}$ in methanol) for 20 min in a dark room. Then the slides were washed in phosphate-buffered saline (PBS) pH 7.4. The Permeability was accomplished by treating with 0.2% Triton X-100 and 0.2% sodium citrate for 5 min on ice. After cleaning with PBS, 30 μL of TUNEL reaction reagent (Roche, USA) was placed to each sample, they were incubated for 1 hour at 38°C in a humid dark box. Next, after rinsing sufficiently with PBS and examination with fluorescence microscope under 100× magnification (33, 34), the nuclei of sperm cells with fragmented DNA (TUNEL+) indicated bright green color, while the nuclei of the normal cells (TUNEL-) presented pale green color.

### Ethical consideration

All animal testing protocols were performed under the management of the Ethics Committee of the Shahid Sadoughi University of Medical Sciences (Code: IR.SSU.MEDICINE.REC.1396.239).

### Statistical analysis

The results were analyzed using the SPSS software (Statistical Package for the Social Sciences, version 20.0, SPSS Inc., Chicago, Illinois, USA). Means were reported as mean ± standard deviation. One-way ANOVA was applied to evaluate the data, and LSD post-test was performed to determine the difference between the two groups. Two-sided p < 0.05 indicated a statistically significant difference between sperm evaluations.

## 3. Results

### Assessment of sperm parameters

**Figure 1:**
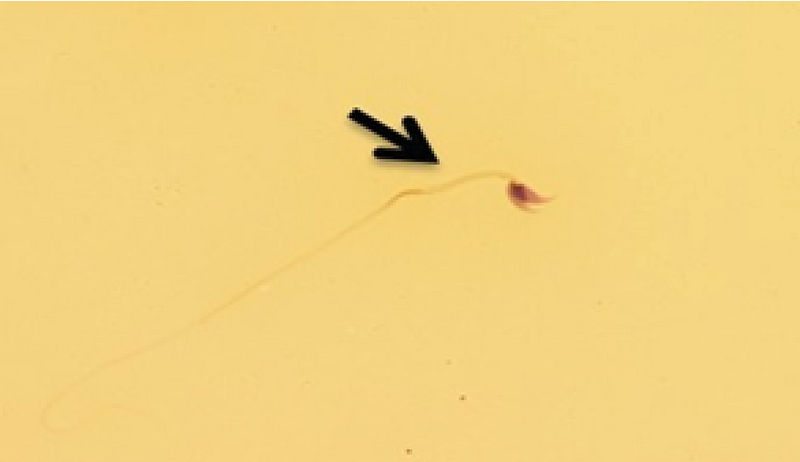
A different form of sperm morphological abnormalcy in the formalin group. The arrow shows abnormal spermatozoon. Diff Quick staining (×100 eyepiece magnification).

While the sperm count displayed a significant decrease in the formalin group in comparison with the other groups (p ≤ 0.001) it was significantly enhanced in the formalin + LC group in comparison with the other study groups (p ≤ 0.001) Table I. with regard to the sperm motility evaluation, progressive motility showed a significant decrease in the formalin group compared to other study groups, while it was significantly enhanced in the formalin + LC group in comparison with the formalin group (p = 0.001). Interestingly, no significant difference was observed between the groups with respect to nonprogressive motility (p ≤ 0.05). However, There was a significant enhancement in the immotile sperm in the formalin group compared to other study groups (p = 0.001) Table I. Further, a significant decrease in the sperm viability was observed in the formalin group in comparison with other study groups (p ≤ 0.001), while it was significantly enhanced in the formalin + LC group in comparison with the formalin group (p ≤ 0.001) Table I. Moreover, while a significant decrease was seen in the normal morphology of the sperm in the formalin group compared to the other groups, a significant rise in the normal morphology of sperm in the formalin + LC group compared to the formalin group was noted (p = 0.001, Figure 1, Table I).

### Chromatin quality and sperm apoptosis assessment

**Figure 2:**
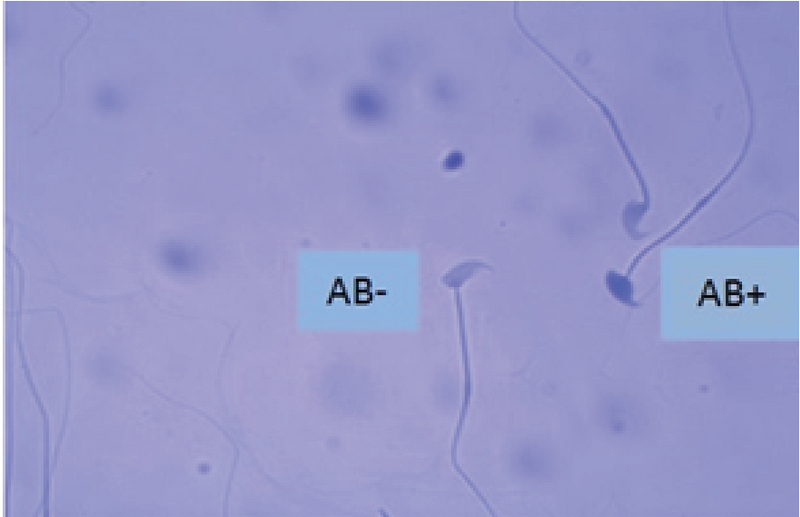
One Aniline Blue-reacted sperm (AB+) and two normal sperm cell (AB-) in the formalin + LC group. Aniline Blue staining (×100 eyepiece magnification).

**Figure 3:**
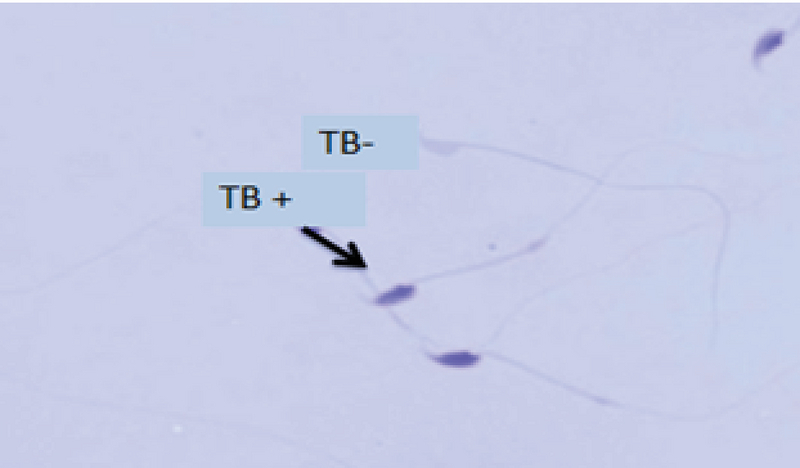
Toluidine blue staining of sperm. (TB+) indicates sperm cells with unusual chromatin and (TB-) displays sperm cells with normal chromatin in the formalin group (×100 eyepiece magnification).

**Figure 4:**
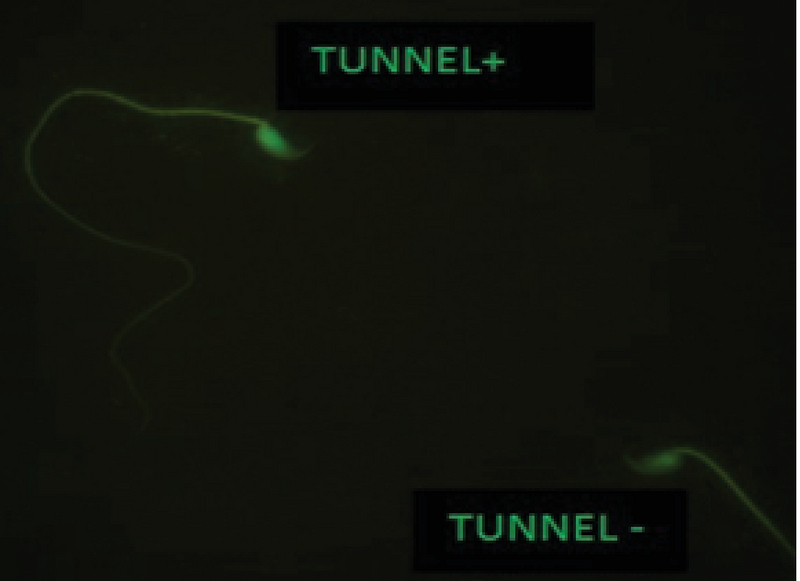
TUNNEL test, Brilliant Apoptotic sperm cell (TUNEL+) and normal sperm cell (TUNEL-) in the formalin + LC group (Fluorescence Microscope ×100).

**Figure 5:**
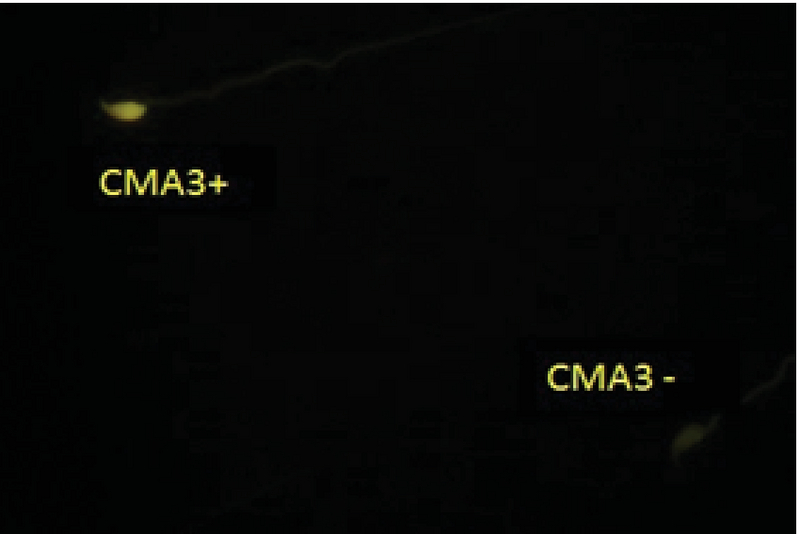
Sperm with protamine insufficiency (CMA3+) and sperm with good protamine amount (CMA3-) in the formalin group. Chromomycin A3 staining (×100 eyepiece magnification).

As seen in Table II, the sperm chromatin integrity and apoptosis, the rates of spermatozoa with AB+, TB+, CMA3+, TUNEL+ showed a significant increase in the formalin group in comparison with the other groups, however it was significantly decreased in the formalin + LC group in comparison with the formalin group (Figures 2-5).

**Table 1:** The results of semen analysis in different groups


**Variables**	**Control (group I)**	**Formalin (group II)**	**Formalin + L-carnitine (group III)**	**P-value**
**Sperm count (10${}^{6}$)**	26.38 ± 2.98	15.52 ± 3.83	32.62 ± 1.04	0<001${}^{abc*}$
**Progressive motility (%)**	52.25 ± 10.29	27.75 ± 12.3	57.25 ± 13.04	0.001${}^{c*}$
**Non progressive motility (%)**	23.5 ± 3.81	20.5 ± 4.3	23.25 ± 4.33	0.002${}^{a*}$
**Immotile sperm (%)**	24.25 ± 8.81	51.75 ± 13.95	19.62 ± 9.63	0<001${}^{ac*}$
**Normal morphology (%)**	61.88 ± 5.22	35 ± 10.5	55.25 ± 9.96	0.001${}^{ac*}$
**Viability (%)**	80.57 ± 8.63	50.71 ± 7.36	83.85 ± 6.2	0<001${}^{ac*}$
Data presented as Mean ± SD, one-way ANOVA test. The mean difference was significant at the 0.05 level. ${}^{a}$ Difference between the control and formalin groups, ${}^{b}$ Difference between the control and formalin + LC groups, ${}^{c}$ Difference between the formalin and formalin + LC groups				

**Table 2:** The results of sperm chromatin/DNA assessment and apoptosis in different groups


**Variables**	**Control (group I)**	**Formalin (group II)**	**Formalin + L-carnitine (group III)**	**P-value**
**Aniline blue (AB) + (%)**	9.62 ± 3.46	55.75 ± 6.11	43.5 ± 6.65	0<001${}^{ab*}$ 0.002${}^{c*}$
**Toluidine blue (TB) + (%)**	18.25 ± 6.94	62.12 ± 5.46	48.88 ± 5.16	0<001${}^{a*}$ 0.004${}^{b*}$ 0.018${}^{c*}$
**Chromomycin A3 (CMA3) + (%)**	6.38 ± 3.37	20.75 ± 4.26	14.38 ± 4.68	0.001${}^{ab*}$ 0.03${}^{c*}$
**TUNEL+ (%)**	2.38 ± 0.91	15.25 ± 2.81	5.62 ± 2.87	0<001${}^{abc*}$
Data presented as Mean ± SD, one-way ANOVA test. The mean difference was significant at the 0.05 level, ${}^{a}$ Difference between the control and formalin groups, ${}^{b}$ Difference between the control and formalin + LC groups, ${}^{c}$ Difference between the formalin and formalin + LC groups				

## 4. Discussion

Various experimental researches have demonstrated the negative impacts of formalin on the male fecundity index such as sperm parameters (35), however, till date, only a limited number of studies have been conducted to determine the effects of formalin on sperm parameters. It should be noted that the results of this study bring new information about the effects of formalin on chromatin compaction and DNA accuracy in mice. According to our results, a significant decline was observed in the sperm parameters in the formalin treated mice when compared with controls. Moladoust and colleagues reported that all semen parameters were detrimentally effected, with the FA displaying undermost count, motility, and viability. FA impel reactive oxygen species (36). In addition, Aitken and co-workers in their studies stated that generation which propels to lipid peroxidation in sperm membrane; wherefore, reduced sperm motility and enhancement chromatin damage accordingly happen (37). Also, ROS suppress intracellular enzymes and eventually decrease the ATP level results in reduced sperm motility (38). Fukushima and co-workers believed that cell cycle arrest and increased apoptosis level are the outcomes of ROS production which leads to decreased sperm count and viability (39).

Aziz and colleagues showed Sertoli stable cells are also exposed to damage by free radicals; when this occurs, the integrity of the germinal epithelium is impaired, resulting in a decrease in sperm count and anomalies in sperm morphology (40). Baird and co-authors showed that facing FA vapor (10 mg/m3 for 2 wk) may result in a reduction in the epididymis sperm source and motility in mice, this experiment also reported that the functions of the antioxidant enzymes were significantly reduced in the testis of mice facing with FA aspiration in comparison with the normal group. One of the rationales for the decrease in sperm motility may be the ability of FA to pass the blood dam, consequently bringing oxidative stress through augmenting reactive oxygen agents or reducing antioxidant activity in the luminal levels (41). In the work by Kose and co-workers focusing on the impact of FA on generative function in mice, the trial animals were faced with FA vapor (12 ppm/1h) for 40 days and the negative impacts of FA on sperm parameters (count, motility, and morphology) were detected (42). Our work indicated that formalin declined sperm motility. As stated by Alkan and colleagues immotile and atypical sperm are able to generate superoxidase, which has an oxidizing effect can decline the sperm quality like motility (43).

The results of this study also showed that high doses of formalin applied in this experiment lead to genotoxic impairment to mice sperm cells and also confirm other relevant outcomes about the mutagenic impacts of facing with formalin in lab animals (44). Duong and co-authors revealed the fundamental pathway that allows formalin to lead to generative and developmental disorders like chromosome instability, oxidative stress, enzymes dysfunction, apoptosis, and interfere with DNA methylation (45). Similar outcomes were obtained by Yoshikawa and co-workers, the sperm quantity in the FA-treated mice declined (50%) in comparison to the normal group in their study (46). In the work by Tang and colleague degradation and destruction to the genetic content of germ cells were detected in male mice facing with doses of 0.3, 3, and 30 mg/kg FA, injected IP for five days. The highest pathological alterations consisting of the decadence of testicular tissue, declined sperm quantity, and morphological alterations in the sperm head were detected in the those facing formalin (47). Expanding male fecundity problems, which is indicated by decrease in sperm condensation and the motility of spermatozoa, have been presented in Western countries (48).

Novel studies have demonstrated the important role of vitamins, nutrients, and minerals in sperm health (49). LC is available in high concentration in the epididymis and plays an important role in the development, maturation and metabolism of spermatozoa by amending sperm motility and exhibiting antioxidant and anti apoptotic confidants for the confirmation of the spermatozoa cell membrane (50). Similar to the results of this study, Banihani and colleagues reported that LC (0.6-1.2 mg /ml) raises human sperm motility and viability when the sperm is incubated at 38°C (51). Similar progress in sperm motility in vitro has been revealed after pouring acetyl- L-carnitine (ALC) into human semen at 38°C (52). This useful impact of LC on human sperm feature is owing to its antioxidant activity and also its role in the sperm metabolism pathway (18). LC, in addition to spermatozoa, reduces oxidative stress and destruction to DNA (53). Prevention of apoptosis via LC has been described in the culture medium of neuronal cells (54). LC increases the function of the DNA-repairing enzyme and other associated repair pathways (55). In this experiment, we proved that the quantity of apoptosis in sperm remarkably augmented in the formalin group in comparison with the normal group. Using LC combined with formalin shrinks the apoptosis in sperm, proposing that LC is able to preserve sperm from formalin damage. Subsequently, the outcomes of this study indicate that LC can be useful in increasing spermatogenesis and fertility in exposure to formalin by decreasing apoptosis. However, more research and experimental studies are needed to diagnose the right pathway of LC impacts in the sperm.

## 5. Conclusion

LC has improving effects on sperm parameters and chromatin density and can reduce the rate of apoptosis in spermatozoa. However, formalin, which is widely used in various industrial fields these days, can adversely effect the reproductive system. We can recommend the use of LC for those who are exposed to these harmful effects of formalin.

##  Conflict of Interest

The authors declare no conflict of interest.
